# Rice Intake Is Associated with Longer Reaction Time and Interacts with Blood Lipids and Hypertension among Qatari Adults

**DOI:** 10.3390/life13010251

**Published:** 2023-01-16

**Authors:** Arwa Elrahmani, Farah Youssef, Haidi Elsayed, Nada Mohamed, Tahra El-Obeid, Zumin Shi

**Affiliations:** Human Nutrition Department, College of Health Sciences, QU Health, Qatar University, Doha 2713, Qatar

**Keywords:** cognition, rice intake, adults, Qatar Biobank study

## Abstract

We aimed to assess the association between rice intake and cognitive function among Qatari adults and test the interactions with health conditions. Data from 1000 adults aged ≥18 years old who attended the Qatar Biobank (QBB) study were used. Rice dietary intake was measured by a food frequency questionnaire (FFQ), and mean reaction time (MRT) was used as an indicator of cognitive function. Linear regression and structure equation models were used. The mean rice consumption was 7.6 times/week (SD 2.0). The sample had a mean MRT of 717 milliseconds (SD 205). Rice consumption was positively associated with MRT. Across the quartiles of rice intake, the regression coefficients (95% CI) for MRT were 0.0 (reference), 22.4 (−7.8, 52.6), 36.3 (5.1, 67.5), and 34.5 (2.6, 66.4). There was a significant interaction between rice intake and hypertension, BMI, and blood lipids in relation to MRT. The association between rice intake and MRT was only observed among those with hypertension, overweight/obesity, low LDL, and low total cholesterol levels. Serum magnesium did not mediate the association. High rice consumption was associated with a higher MRT, especially among those with hypertension, overweight/obesity, low LDL, and or low total cholesterol levels. Further longitudinal studies are needed to confirm the findings.

## 1. Introduction

Reaction time (RT) is a measure of processing speed or efficiency in the central nervous system [1]. It is a major determinant of higher cognitive function [2]. In addition to older age, men, lower education, and smoking are also associated with RT in some population studies [3]. Population studies suggest that diet is one of the important determinants for cognition function [4,5,6].

Rice is the main staple food for almost two billion people (~50%) in Asia [7,8]. The association between rice consumption and health outcomes has been examined in a limited number of studies with inconsistent findings [9,10,11,12]. In Asian populations specifically, two meta-analyses found that higher rice consumption is associated with an increased risk of type 2 diabetes [9,10]. While rice consumption increases the risk of diabetes and abnormal blood lipids in some populations, rice consumption has been shown to be inversely associated with obesity, hypertension, and cardiovascular diseases in other studies [11,12]. Chronic diseases such as diabetes and hypertension are associated with a higher risk of mild cognitive impairment (MCI) [13]. Only two population studies have examined the association between rice intake and cognition [14,15]. Studies in China found that higher rice intake was associated with a higher likelihood of functional impairment, difficulties in decision making, and a decline in cognitive function [14,15]. In the Shanghai Women’s Health Study and the Shanghai Men’s Health Study, those with high rice intake were over 20% more likely to have memory problems [14]. A 2-year cohort study conducted in the Chinese population concluded that weekly higher white rice intake was directly associated with an increased risk of incident mild cognitive impairment (MCI) [15]. In the study, a total of 471 participants without cognitive impairment at baseline developed incident MCI during the two-year follow up. High white rice consumption was associated with an increased risk of MCI (HR = 1.051, 95% CI: 1.008~1.096) and was independent of education, age, and drinking [15]. A randomized clinical trial that recruited 52 Japanese elderly participants aged 65 and above (25 men and 27 women) showed that the group that consumed white rice required more time to answer questions in the computerized cognitive function test compared with the brown rice group. Furthermore, the mean change in the total time required to answer all questions of the test was shorter in the brown rice group than in the white rice group [16]. However, the association between rice consumption and cognition has not been studied in countries with different food cultures. The mechanisms linking rice intake and cognition have not been tested. Furthermore, there is a lack of studies on the interaction between rice intake and chronic diseases. Blood lipids are associated with cognitive function. While higher total cholesterol and low-density lipoprotein cholesterol (LDL-C) are related to a higher risk of Alzheimer’s disease in the 3C study [17], high-density lipoprotein cholesterol (HDL-C) and triglycerides were positively associated with cognitive function in overweight and obese individuals in China [18].

In Qatar, rice is the staple food [19]. Data from the Qatari Planning and Statistics Authority showed that the mean household purchase of rice was 56 kg/month [20], which is comparable to the consumption level in China [12]. The high consumption of rice among the Qatari population is partly because it is part of the subsidized monthly food rations, which also include oils, condensed milk, and sugar. Expenditure on rice was 49% of the cereals category in the food basket composition of Qatari households [19].

Refined grain (such as rice) consumption has been shown to contribute to a low magnesium consumption [21]. In many countries, magnesium consumption decreased over the past decades due to the low consumption of whole grains and fresh fruits and vegetables. Low serum magnesium is related to a poor cognitive function in Qatari adults [22]. It is unknown whether low serum magnesium mediates the association between rice intake and cognitive function.

To address the above knowledge gap, the aims of the study were to (1) assess the association between rice consumption and cognitive function among Qatari adults; (2) test the interaction between rice intake and hypertension, BMI, diabetes, and blood lipids; and (3) test whether serum magnesium mediates the association between rice intake and cognition. We have three hypotheses: (1) rice intake was positively associated with reaction time; (2) there was an interaction between rice intake and chronic diseases; and (3) magnesium mediated the association between rice intake and reaction time.

## 2. Materials and Methods

### 2.1. Study Design and Study Sample

Data from 1000 (500 men and 500 women) randomly selected Qatari adults aged 18 years old and above who attended the Qatar Biobank study (QBB) were analyzed. The details of the QBB study have been published elsewhere [23]. In short, Qatari adults or long-term residents (aged ≥18 years) were invited to participate in the study with a goal to include 60,000 participants, with follow ups planned for every 5 years. To be eligible for the analysis, participants were required to have data on food intake and attend the cognition test. By using a self-administered questionnaire, the sociodemographic data, dietary habits, and lifestyle factors were obtained. The biomarkers in the QBB cohort consisted of 66 clinical biomarkers which were routinely measured on the participants’ blood samples [24]. The sample size in the current analysis was determined by the fact that QBB provides 1000 samples to research projects conducted at Qatar University for free. This study was conducted according to the guidelines in the Declaration of Helsinki and all procedures involving human subjects/patients were approved by the Hamad Medical Corporation Ethics Committee in 2011 and continued with the QBB Institutional Review Board from 2017 onward. Written informed consent was obtained from all subjects/patients. For the current study, the ethical approval was obtained from QBB (Ex-2021-QF-QBB-RES-ACC-00035-0164).

### 2.2. Outcome Variable: Cognitive Function (Mean Reaction Time)

Mean reaction time (MRT) was used as an indicator for cognitive function. The MRT test is a computer-based, self-administered touch screen test and consists of 60 tasks using a visual stimulus [24,25]. It was designed by the Cambridge Neuropsychological Test Automated Battery (CANTAB) (https://www.cambridgecognition.com/cantab, accessed on 27 December 2022). The task generates 60 presentations of one of two targets. The target is presented as a small white box within one of two larger black boxes. The location of the target within the black box varies. During each trial, the participant has to select the box where the target appears as quickly as possible. The reaction time each individual took to complete each of the 60 tasks was measured. The mean value of the reaction time of the 60 tasks was calculated and used in the current study. A higher MRT represents a worse cognitive function. MRT was used to measure cognitive function in population studies [25,26].

### 2.3. Exposure Variable: Rice Intake

Rice dietary intake was assessed by a food frequency questionnaire (FFQ) that was self-administered and computerized. The participants were asked about their intake frequency as well as their dietary habits [23]. The FFQ contained 102 food items and was adapted from the European Perspective Investigation into the Cancer and Nutrition (EPIC) study; however, it has not been validated in Qatar. However, the food items included in the FFQ were similar to a recent validated FFQ in Qatar [27].

### 2.4. Covariates

The following covariates were used in the study: gender, age, BMI (categorical, overweight 25.0–29.9 kg/m^2^ and obesity ≥30 kg/m^2^), level of education (below or above university education), medication use, smoking (non-smokers, ex-smokers, and current smokers), intake of fruit and vegetable, total leisure time physical activity level (MET hours/week) [28], and self-reported chronic conditions. Fruit and vegetable intake (times/week) were self-reported. The criteria for diagnosing diabetes were: HbA1c ≥ 6.5%, random blood glucose (RBG) of ≥11.1 mmol/L, fasting blood glucose (FBG) of ≥7 mmol/L, or self-reported diabetes [29]. Hypertension was defined as systolic blood pressure ≥140 mmHg or diastolic is ≥90 mmHg or previous doctor diagnosis. Total cholesterol, HDL-C, and triglyceride levels were measured using standard laboratory enzymatic methods. LDL-C was calculated using the Friedewald formula [30]. Serum magnesium was measured by an automated colorimetric method (Magnesium Gen. 2 from Roche Diagnostics, Indianapolis, IN, USA) [22].

### 2.5. Statistical Analysis

Rice intake was categorized into quartiles. Sample characteristics were presented as percentage or mean (SD). Chi-squared test and ANOVA were used to test the differences in continuous variables and categorical variables by quartiles of rice intake. For our research aim 1, three multiple linear regression models were used to examine the association between rice intake and MRT. Model 1 was adjusted for gender and age. Model 2 was further adjusted for smoking, education, fruit and vegetable intake, and physical activity. Model 3 was further adjusted for BMI (continuous), hypertension, diabetes, and medication use. The variables adjusted were either sociodemographic factors or known risk factors for cognitive impairment. For our research aim 2, by adding the product terms of the two variables in the linear regression model, the interactions between rice intake, chronic diseases (diabetes and hypertension), BMI, and blood lipids were tested in the corresponding multivariable model. The interaction was visualized using the marginsplot command. For our research aim 3, the structural equation model was used to test the direct and indirect effect (via serum magnesium) of rice on cognitive function. STATA (Version 17, Stata Corporation, College Station, TX, USA) was used for all the analysis. *p*-values < 0.05 (2-tailed) were considered significant.

## 3. Results

### 3.1. Sample Characteristics

The mean age of the sample taken was 35.8 (SD 10.4) years (Table 1). More than half of the participants had a high educational level (65.9%). Most of the participants were non-smokers (67.1%), while smokers made up 18.8% of the sample, and 14.1% were former smokers. The mean BMI was 28.2 (SD 5.7) kg/m^2^. The majority of the participants were either overweight or obese, making up almost 70.5% of the sample. The sample had a mean MRT of 717 (SD 205) milliseconds. The mean serum magnesium was 0.84 (SD 0.06) mmol/L.

The mean rice consumption was 7.6 times/week, ranging from 1.8 times/week in quartile 1 (Q1) to 15.1 times/week in quartile 4 (Q4). Across the quartiles of rice intake, the intake of fruits and vegetables increased but the education level decreased. The prevalence of diabetes was higher in the high rice consumption group compared with low intake (16.5% vs. 10.5%). There was no difference in age, smoking, physical activity, BMI, and hypertension across the quartiles of rice intake.

### 3.2. Association between Rice Consumption and MRT

Rice consumption was directly associated with a higher MRT (Table 2). After adjusting for age, gender, and lifestyle factors, the regression coefficients for MRT were 0.0 (reference), 22.4 (−7.8, 52.6), 36.3 (5.1, 67.5), and 34.5 (2.6, 66.4) across the quartiles of rice consumption. After further adjusting for BMI, diabetes, and hypertension, the above association was attenuated and became borderline significant. The association was independent of serum magnesium level (model 4). In the structure equation model, there was no mediating effect by serum magnesium (Table 3). Excluding participants with extreme values of MRT (i.e., <1st or >99th), the above findings remained.

### 3.3. Interactions between Rice Intake and Chronic Conditions

**Figure 1:**
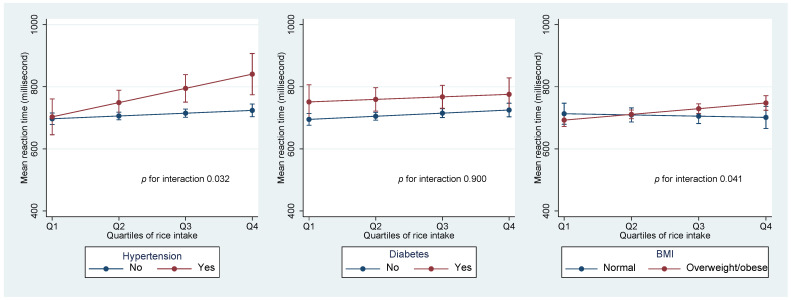
Interaction between rice intake and hypertension, diabetes, and BMI in relation to MRT.

There was a significant two-way interaction (*p* = 0.032) between hypertension and rice in relation to cognitive function. High consumption of rice was associated with higher MRT among those with hypertension (Figure 1). However, no interaction between rice consumption and diabetes was found.

A significant interaction (*p* = 0.041) was found between rice intake and BMI in relation to MRT. No association between rice intake and MRT was found among people who had a normal BMI. However, among those with overweight/obesity, higher rice intake had a higher MRT.

Values were marginal means (SD). Models were adjusted for age, gender, education, smoking, leisure time and physical activity, and intake of fruit and vegetable.

**Figure 2:**
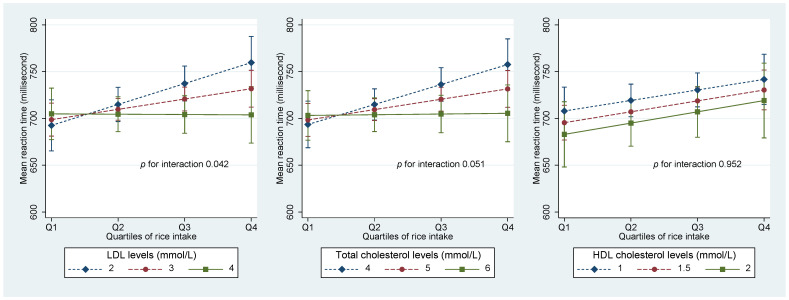
Interaction between rice intake and blood lipids in relation to MRT. Values were marginal means (SD). Models were adjusted for age, gender, education, smoking, leisure time and physical activity, and intake of fruit and vegetable. Values for LDL, HDL, and total cholesterol presented were around 10, 50, and 90 percentiles.

LDL cholesterol level had a significant interaction with rice intake in relation to MRT. Figure 2 shows that among people with a low LDL level, there was a positive association between rice intake and MRT. However, there was no similar association among those with a high LDL. A borderline significant (*p* = 0.051) interaction was found with total cholesterol levels. However, no such interaction was found with HDL levels.

## 4. Discussion

In this cross-sectional study, we found that rice consumption is positively associated with MRT. There was an interaction between rice consumption and hypertension, BMI, LDL, and total cholesterol levels in relation to MRT. The positive association between rice intake and MRT was only observed among those with hypertension, high BMI, low LDL, and low total cholesterol levels.

Most of the studies on rice consumption and cognitive function were conducted in Asian populations [14,15,16,31]. Direct comparison between our study and other population studies is difficult due to the difference in the outcome measures. Our findings on the positive association between rice consumption and MRT are in line with other studies [14,15,16,31].

### 4.1. Potential Mechanisms

Several mechanisms may explain the link between rice intake and cognitive function impairment including low magnesium levels in refined rice, the positive association between rice and diabetes, the high glycemic index, and heavy metal contamination.

Inadequate micronutrients (e.g., magnesium) may also explain the link. Magnesium is an important nutrient that can affect cognition [24]. Magnesium is found in limited amounts in refined grains; and the Qatari dietary pattern is characterized by high consumption of refined grains such as rice and wheat [24]. A study in Qatar showed that there is an association between the concentrations of serum magnesium and cognitive function. It suggested that low serum magnesium is associated with increased MRT especially in women with diabetes and hypertension [24]. However, in our study, serum magnesium is not a mediator between rice intake and cognitive function. Further research is needed to validate the finding.

Evidence from meta-analyses suggests that high rice consumption increases the risk of diabetes in Asian populations [9,10]. It has been shown that diabetes increases the risk of cognitive function impairment [32]. Rice, especially refined white rice, has a high glycemic index. Based on a systematic review, consumption of a low glycemic index diet may favor cognitive function in adults [33]. High consumption of a high-glycemic-index rice-based diet may increase the risk of stroke in some populations [34]. It is well known that stroke increases the risk of cognitive impairment. The prevalence of post-stroke cognitive impairment is estimated to range from 20% to 80% [35].

The interaction between rice intake and hypertension may be because hypertension is a risk factor for cognitive impairment. For example, in a cross-sectional study of Chinese adults aged 60 years and above (n = 46,011), hypertension was associated with both dementia (OR 1.86 (95% CI 1.70–2.03) and mild cognitive impairment (MCI) (OR 1.62 (1.54–1.7) [36].

The interaction between blood lipids and rice intake is intriguing. The mechanisms are yet to be studied. A study involving 789 men and 1105 women from the Framingham Heart Study cohort indicated a positive direct association between total cholesterol (TC) and cognitive measures such as attention, abstract reasoning, and several cognitive domains [37]. It could be due to the need of TC for neural cells to perform normal metabolic processes [38]. Some studies suggested that high TC in older adults is associated with improved cognitive function [37,38]. It would be possible that under the condition of low cholesterol level, high rice consumption aggravates the impairment of cognitive function.

Oxidative stress (OS) is a main factor contributing to cognitive impairment as it has a role in the pathophysiology and neuronal degeneration that leads to diseases such as dementia, mild cognitive impairment (MCI), and eventually Alzheimer’s disease [39]. Rice consumption may increase oxidative stress by various mechanisms including reducing liver function [40] and increasing the risk of non-communicable chronic diseases.

The bioaccumulation of arsenic in rice and the subsequent consumption by consumers can lead to adverse health effects [41]. Chronic arsenic exposure decreases serum brain-derived neurotrophic factor (sBDNF) and cognitive function in adults [42].

Finally, rice-related dietary patterns may also explain the link. Based on Qatari cuisine, foods high in saturated fat such as lamb meat are often eaten as an accompaniment to rice [19]. In Qatar, rice is also consumed as a dessert with high amounts of sugar and ghee added to it. A cross-sectional study on middle-aged adults found that intake of saturated fat was associated with an increased risk of cognitive function impairment [43].

### 4.2. Strengths and Limitations

This research has several strengths. Firstly, the participants were selected from Qatar’s general population using data from the Qatar Biobank study. Secondly, we were able to adjust for many confounding variables. Thirdly, the large variation in rice intake in the sample allowed us to examine the association with cognitive function. However, there are several limitations. First, MRT was the only outcome measurement used to assess cognition, thus it could not represent other aspects of cognition. As QBB has started to conduct MRI brain scans, in the future we will be able to examine the relationship between rice intake and brain images. Second, we have information on the frequency of rice intake but not the absolute intake. However, the frequency of intake may reflect the eating habit. Third, the study lacks comprehensive information on medication use. Moreover, due to its cross-sectional research methodology, the study cannot indicate a cause–effect link. In the study, a high intake of rice was associated with a low education level. Although we have adjusted for education, residual confounding is possible. Furthermore, we do not have information on heavy metal levels in the blood or urine. Future studies should measure heavy metals in blood or urine and examine whether heavy metals mediate the association between rice intake and cognitive function. Cohort studies are warranted to validate our exploratory findings.

## 5. Conclusions

In conclusion, rice consumption was directly associated with cognitive function as measured by the mean reaction time. High rice consumption was associated with a higher MRT among those with hypertension, overweight/obesity, low LDL, and low total cholesterol levels. Further studies are needed to validate the findings.

## Figures and Tables

**Table 1:** Sample characteristics by quartiles of rice consumption among participants attending the Qatar Biobank study (N = 1000).

	Q1 (n = 306)	Q2 (n = 244)	Q3 (n = 219)	Q4 (n = 231)	*p*-Value *
Rice intake (times/week)	1.8 (1.0)	5.2 (0.8)	8.1 (0.9)	15.1 (5.4)	<0.001
Age (years)	36.1 (9.6)	35.4 (11.0)	36.9 (10.8)	34.8 (10.0)	0.167
Gender					0.581
Male	147 (48.0%)	117 (48.0%)	115 (52.5%)	121 (52.4%)	
Female	159 (52.0%)	127 (52.0%)	104 (47.5%)	110 (47.6%)	
Education					0.001
Low (below university)	94 (30.7%)	73 (30.0%)	68 (31.1%)	103 (44.8%)	
High (university or above)	212 (69.3%)	170 (70.0%)	151 (68.9%)	127 (55.2%)	
Smoking					0.785
Non	213 (69.6%)	162 (66.4%)	145 (66.2%)	153 (66.2%)	
Smoker	52 (17.0%)	45 (18.4%)	40 (18.3%)	50 (21.6%)	
Ex-smoker	41 (13.4%)	37 (15.2%)	34 (15.5%)	28 (12.1%)	
Leisure time physical activity (MET hours/week)	5.8 (17.9)	5.2 (15.0)	6.3 (17.0)	7.9 (35.5)	0.598
BMI (kg/m^2^)	28.8 (5.6)	27.8 (5.5)	28.0 (6.0)	28.1 (5.8)	0.212
BMI categories					0.784
Normal	79 (25.8%)	76 (31.1%)	67 (30.6%)	71 (30.7%)	
Overweight	118 (38.6%)	93 (38.1%)	83 (37.9%)	88 (38.1%)	
Obese	109 (35.6%)	75 (30.7%)	69 (31.5%)	72 (31.2%)	
Supplement use	189 (61.8%)	158 (64.8%)	141 (64.4%)	126 (54.5%)	0.087
Vitamin D and calcium use	119 (38.9%)	105 (43.0%)	85 (38.8%)	74 (32.0%)	0.101
Vegetable intake (times/week)	14.2 (12.0)	15.3 (11.5)	17.5 (11.8)	23.1 (18.1)	<0.001
Fruit intake (times/week)	5.7 (5.5)	6.9 (6.4)	7.1 (6.0)	7.9 (6.8)	<0.001
Magnesium (mmol/L)	0.84 (0.05)	0.84 (0.06)	0.83 (0.06)	0.83 (0.06)	0.046
LDL (mmol/L)	3.0 (0.8)	3.0 (0.8)	2.9 (0.8)	2.9 (0.9)	0.461
HDL (mmol/L)	1.4 (0.4)	1.4 (0.4)	1.3 (0.4)	1.3 (0.4)	0.322
Total cholesterol (mmol/L)	5.0 (0.9)	4.9 (0.9)	4.9 (0.9)	4.9 (0.9)	0.697
HbA1C (%)	5.5 (0.9)	5.5 (0.8)	5.5 (0.8)	5.7 (1.1)	0.137
Hypertension	30 (9.8%)	20 (8.2%)	29 (13.2%)	17 (7.4%)	0.154
Diabetes	31 (10.5%)	25 (10.5%)	24 (11.3%)	36 (16.5%)	0.149
Insulin use	4 (1.3%)	3 (1.2%)	4 (1.8%)	8 (3.5%)	0.241
Diabetes medication other than insulin	8 (2.6%)	14 (5.7%)	17 (7.8%)	16 (6.9%)	0.046
Hypertension medication use	12 (3.9%)	11 (4.5%)	18 (8.2%)	14 (6.1%)	0.159
Mean reaction time (millisecond)	693.72 (175.83)	711.85 (194.49)	734.41 (250.92)	729.35 (197.96)	0.089

* One-way ANOVA was used for continuous variables; Chi-square test was used for other categorical variables listed in the table.

**Table 2:** Association between quartiles of rice intake and cognitive function as measured by mean reaction time.

	Q1 (n = 306)	Q2 (n = 244)	Q3 (n = 219)	Q4 (n = 231)	*p* for Trend
Model 1	Ref	23.6 (−7.0, 54.1)	38.6 (7.2, 70.1)	50.1 (19.0, 81.1)	0.001
Model 2	Ref	22.4 (−7.8, 52.6)	36.3 (5.1, 67.5)	34.5 (2.6, 66.4)	0.017
Model 3	Ref	17.1 (−13.5, 47.8)	26.6 (−5.3, 58.4)	26.3 (−6.5, 59.0)	0.079
Model 4	Ref	21.9 (−8.3, 52.1)	33.7 (2.4, 65.0)	33.0 (1.1, 64.9)	0.024

Values are regression coefficients (95% CI) from linear regression. Model 1 adjusted for age and gender. Model 2 further adjusted for education, smoking, physical activity, fruit, and vegetable intake. Model 3 further adjusted for BMI, hypertension, and medication use for diabetes and hypertension. Model 4 is Model 2 plus further adjustment for serum magnesium.

**Table 3:** Direct and indirect effect of rice intake on cognitive function measured by mean reaction time.

	β (95% CI)	*p* Value
Total effect	3.13 (1.13–5.12)	0.002
Direct effect	3.00 (1.01–5.00)	0.003
Indirect effect (via serum magnesium)	0.13 (−0.05–3.09)	0.160

Model was adjusted for age and gender. Rice intake was modeled as a continuous variable in the structure equation model analysis.

## Data Availability

The data can be requested from the Qatar Biobank study data management team.
